# Account of Diseases in an Eastern District of London, from January 20, to February 20, 1805

**Published:** 1805-03-01

**Authors:** 


### *Account of Diseases in an Eastern District of London, from January 20, to February 20, 1805.*

| | |
|---|---|
| **ACUTE DISEASES.** | |
| Pneumonia | 3 |
| Peripneumonia Notha | 5 |
| Scarlatina | 2 |
| Rheumatismus Acutus | 6 |
| **CHRONIC DISEASES.** | |
| Tussis | 20 |
| Tussis cum Dyspnœa | 32 |
| Dyspnœa | 15 |
| Phthisis Pulmonalis | 3 |
| Dyspepsia | 4 |
| Ascites | 2 |
| Hydrothorax | 3 |
| Menorrhagia | 7 |
| Amenorrhœa | 8 |
| Leucorrhœa | 9 |
| Paralysis | 2 |
| Diarrhœa | 12 |
| Rheumatismus Chronicus | 20 |
| **PUERPERAL DISEASES.** | |
| Convulsio | 1 |
| Menorrhagia Lochialis | 6 |
| Enteritis | 1 |
| Ephemera | 5 |
| **INFANTILE DISEASES.** | |
| Ophthalmia Purulenta | 2 |
| Vermes | 4 |
| Aphthæ | 3 |
| Erysipelas Infantile | 2 |

MEDICAL

